# Genetic signature of the natural gene pool of *Tilia cordata* Mill. in Lithuania: Compound evolutionary and anthropogenic effects

**DOI:** 10.1002/ece3.7473

**Published:** 2021-05-01

**Authors:** Darius Danusevičius, Rūta Kembrytė, Jurata Buchovska, Virgilijus Baliuckas, Darius Kavaliauskas

**Affiliations:** ^1^ Vytautas Magnus University Kaunas Lithuania; ^2^ Forestry Institute Lithuanian Research Centre for Agriculture and Forestry Kaunas Lithuania; ^3^ Bavarian Office for Forest Genetics (AWG) Teisendorf Germany

**Keywords:** gene flow, genetic diversity, genetic structure, inbreeding, microsatellites, mixed forests, riparian, small‐leaf lime, species expanding

## Abstract

*Tilia cordata* Mill. is a valuable tree species enriching the ecological values of the coniferous‐dominated boreal forests in Europe. Following the historical decline, spreading of *Tilia* sp. is challenged by the elevated inbreeding and habitat fragmentation. We studied the geographical distribution of genetic diversity of *Tilia cordata* populations in Lithuania. We used 14 genomic microsatellite markers to genotype 543 individuals from 23 wild‐growing populations. We found that *Tilia cordata* retained high levels of genetic diversity (population *F*_IS_ = 0–0.15, *H*_o_ = 0.53–0.69, *H*_e_ = 0.56–0.75). AMOVA, Bayesian clustering, and Monmonier's barrier detection indicate weak but significant differentiation among the populations (*F*_ST_ = 0.037***) into geographically interpretable clusters of (a) western Lithuania with high genetic heterogeneity but low genetic diversity, bottleneck effects, (b) relatively higher genetic diversity of *Tilia cordata* on rich and most soils of midland lowland, and (c) the most differentiated populations on poor soils of the coolest northeastern highland possessing the highest rare allele frequency but elevated inbreeding and bottleneck effects. Weak genetic differentiation among the *Tilia cordata* populations in Lithuania implies common ancestry, absence of strong adaptive gradients, and effective genetic exchange possible mediated via the riparian networks. A hypothesis on riparian networks as gene flow mediators in *Tilia cordata* was raised based on results of this study.

## Graphical abstract

Gene pool of *Tilia cordata* in Lithuania contains the autochthonous populations of high genetic diversity representing the pre‐historical gene pools, that can be promoted, and the escapes from urban sources of low diversity, that must be contained.

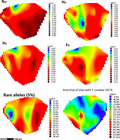

## 1 INTRODUCTION

Warming climate in semiboreal forests promotes spreading of broadleaved tree species leading to natural formations of mixed forest stands (Hampe & Petit, 2005; Hemery et al., 2010). One such species is *Tilia cordata* (Mill.) spreading after its historical retreat into the autochthonous woodlands of northerly Europe during the late Holocene (Balakauskas, 2012; De Jaegere et al., 2016; Phuekvilai, 2014). Here, several fundamental questions relevant to northern forest communities arise. How genetically diverse is the historical gene pool of *Tilia cordata* in the retreats of the northerly forests? What is the genetic structure within a tree species that is largely insect‐pollinated and forecasted to form mixed forests in the future? Is it structured in small isolated pollutions or, on the opposite, as large intermating gene pools as wind‐pollinated species do. Revealing these genetic properties will improve understanding of species ecology and support the natural integration of *Tilia cordata* into the northerly forests (Ennos, 2003; Myking, 2002).

*Tilia cordata* is an interesting species for the population genetic studies because of its turbulent evolutionary history, insect‐mediated reproductive system, and extensive urban use (De Jaegere et al., 2016). Below, we extracted important details on the effects of these three factors on the gene pool of *Tilia* sp. The warm Atlantic period (*ca*. 7000–4000 BC) is considered as the golden age for *Tilia* sp. dominating the forests of northern Europe (Rehfeldt, 1994). Following the cooling temperatures at the later part of Holocene ca. 3000 years BC, frequency of *Tilia cordata* dropped markedly in the northerly forests (Balakauskas, 2012; Hewitt, 2004). In the absence of favorable temperatures at the right time for seed germination and pollination/fertilization, success leads to scattered retreats of *Tilia cordata* into fragmented forest gene pools (Pigott & Huntley, 1981). Owing to a reduced dispersal capacity in a cooler climate, *Tilia cordata* rarely outcompetes the rival pioneers and seldom reoccupies the former habitats (Pigott & Huntley, 1981). In the later centuries, forest clearance for agriculture has further reduced the remaining autochthonous *Tilia* forests in Europe. Provided this retreat of the natural gene pools, the Tilia plantations in avenues “under the linden tree” of urban parks are likely to have served as the escape sources of *Tilia* groups into the wild already since the medieval times (Lefevre, 2004). Therefore, we may deal with compound genetic structures for such species as Tilia: the autochthonous gene pools and introduced populations as the case of European beech in Lithuania (Kembryte et al., 2021). The surviving ancient especially coppice‐born admixture of *Tilia cordata* in European forests is often considered as an indicator of the autochthonous woodlands (Hermy et al., 1999; Lawesson, 2004; Rackham, 2008).

At the ecosystem level, *Tilia cordata* forms biocommunities with added benefits for ecosystem services, including bee conservation (Anderson, 1976; Free, 1970; Pigott & Huntley, 1981). In the forest stands, presence of an admixture of *Tilia cordata* is associated with high diversity of plant species (Normann et al., 2016) and its decomposing leaves enrich soil with nutrients (Muys et al., 1992).

The reproductive system of *Tilia cordata* is dual—where the environment is favorable, it relays on its generative abilities (Anderson, 1976; Pigott, 2012; Tutin et al., 1968), where it turns to harsher conditions, vegetative sprouting prevails, especially when moving northwards (Honnay & Bossuyt, 2005; Knuth, 1908). According to Barker (2017) and Logan et al. (2015), ca. 25%–40% of *Tilia cordata* trees within UK populations originate from vegetative sprouting. In the generative system, the flower nectar production and pollen germination require temperatures above 15°C (Pigott et al., 1980; Tal, 2006). *Tilia* certainly is an entomophilous species, pollinated by mainly bees (daytime) and short tongue moth (nighttime) (Anderson, 1976; Pigott, 1991). Wind is a secondary pollinator in *Tilia*, however, due to the dense crowns with fully extended, the effective wind‐mediated pollen flow reaches fewer than ca. 200 m (Anderson, 1976). Whereas bees may fly up to 6 km away from their nests (Pasquet et al., 2008). Dispersal of mature *Tilia* seed usually is limited to ca. 300 m (Pigott, 2012). The bracted seed can float and be viable for germination after a 30 km travel along the steams (Pigott, 2012). This riparian movement of seeds may serve as a potential gene flow mediator.

Despite the sensitive reproduction system, and environmental and anthropogenic pressure, *Tilia cordata* populations survived over vast territories from Spain to central Sweden in the north and the Ural Mountains in the east (Pigott, 2012). The northern expansion of *Tilia cordata* was limited by the lack of favorable summer temperatures required for successful reproduction (Pigott & Huntley, 1981). In different parts of Europe, these autochthonous gene pools were subjected to diverse adaptive pressure, land‐use history, urban development, and forest use intensity. This led to a marked regional variation in the genetic condition of *Tilia cordata* populations in Europe (Erichsen et al., 2019; Lobo et al., 2018; Logan et al., 2019). For instance, the competitive advantage of European beech is usually referred as an important cause for decline of *Tilia* sp. in Europe (Turner, 1962). However, in most of the present‐day semiboreal and boreal zones, beech was not present.

**FIGURE 1:**
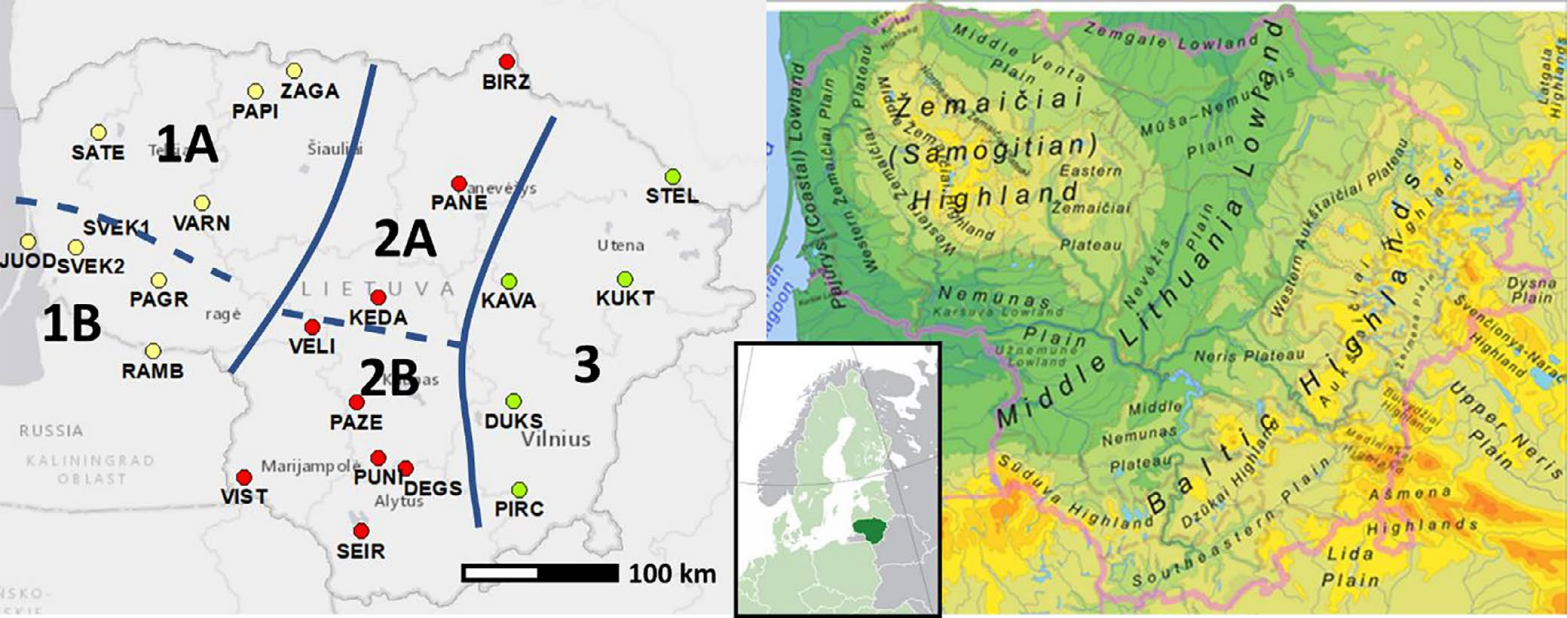
Location map of the populations studied (left). The three main ecoclimatic regions (1, 2, and 3) are separated by the solid line (mainly coastal–continental gradient affected by the altitudinal variation) and the subregions (a, b) reflecting a finer zoning are marked by dashed lines. Population position markers are colored according to the three main regions. Altitudinal gradients in Lithuania (left, the highlands peak to 240–290 m a.s.l.). The northeastern highland (region 3) contains the coolest sites in the country

As regards the eastern Baltic region, the forests are in the transitional zone between the boreal and temperate forests. However, recent climatic shift leads to a steady expansion of mixed broadleaved forests (Pearson & Dawson, 2003). The present‐day forest cover in Lithuania is 33.5%. A large share of these forests is autochthonous, naturally regenerated over centuries, and fits well to the definition of ancient woodland (Peterken, 1974). Eco‐climatically, the country is split into coastal lowland, western highland, midland lowland, and eastern highland (Figure 1). Scots pine (*Pinus sylvestris*) dominates on poor sandy sites in south and southeast. Mixed Norway spruce (*Picea abies*) Scots pine forests are common in the northeastern and western highlands. Midland lowland contains relatively richer soils and is dominated by broadleaved tree species. According to the forest inventory from 2010, the forest sites with over 50% dominance of *Tilia cordata* comprise 6,500 ha in Lithuania, which is 0.32% of the total area of forest land in Lithuanian. *Tilia cordata* is more frequent on rich soils in the forests of midland lowland, especially its southwestern and northern parts (Semaškienė, 2006, Figure 2). Depending on the moisture regime, the forest sites with dominance of *Tilia cordata* are commonly admixed with English oaks (*Quercus robur*), aspen (*Populus tremula*), birch (*Betula pendula*), Norway spruce, hornbeam (*Carpinus betulus*), black and gray alders (*Alnus glutinosa, incana)*, Norway maple (the richer the site, the more diverse the species comparison) (Semaškienė, 2006). Tilia cordata usually forms rather fragmented communities of ca. 5–10 ha at large. These communities can be found deep inside forest tracts as well as at the edges. *Tilia cordata* is also very common tree for landscape amenities and urban areas, such as city avenues, and modern and old manor house parks of over entire eastern Baltic (Tauras, 1989). Especially after the devastation of WW2, there was a campaign for green landscape restoration that encouraged establishment of small parks and planting trees along the streets in small villages, towns, and cities in Lithuania (Tauras, 1989). In many of these post‐WW2 plantations, Tilia cordata was used as a native entomophilous tree with cultural and medical values. Likely seed sources for these trees were old manor house parks (Tauras, 1989). Tilia platyphyllos does not occur naturally in Lithuania but is not rare as a decorative tree in urban parks.

**FIGURE 2:**
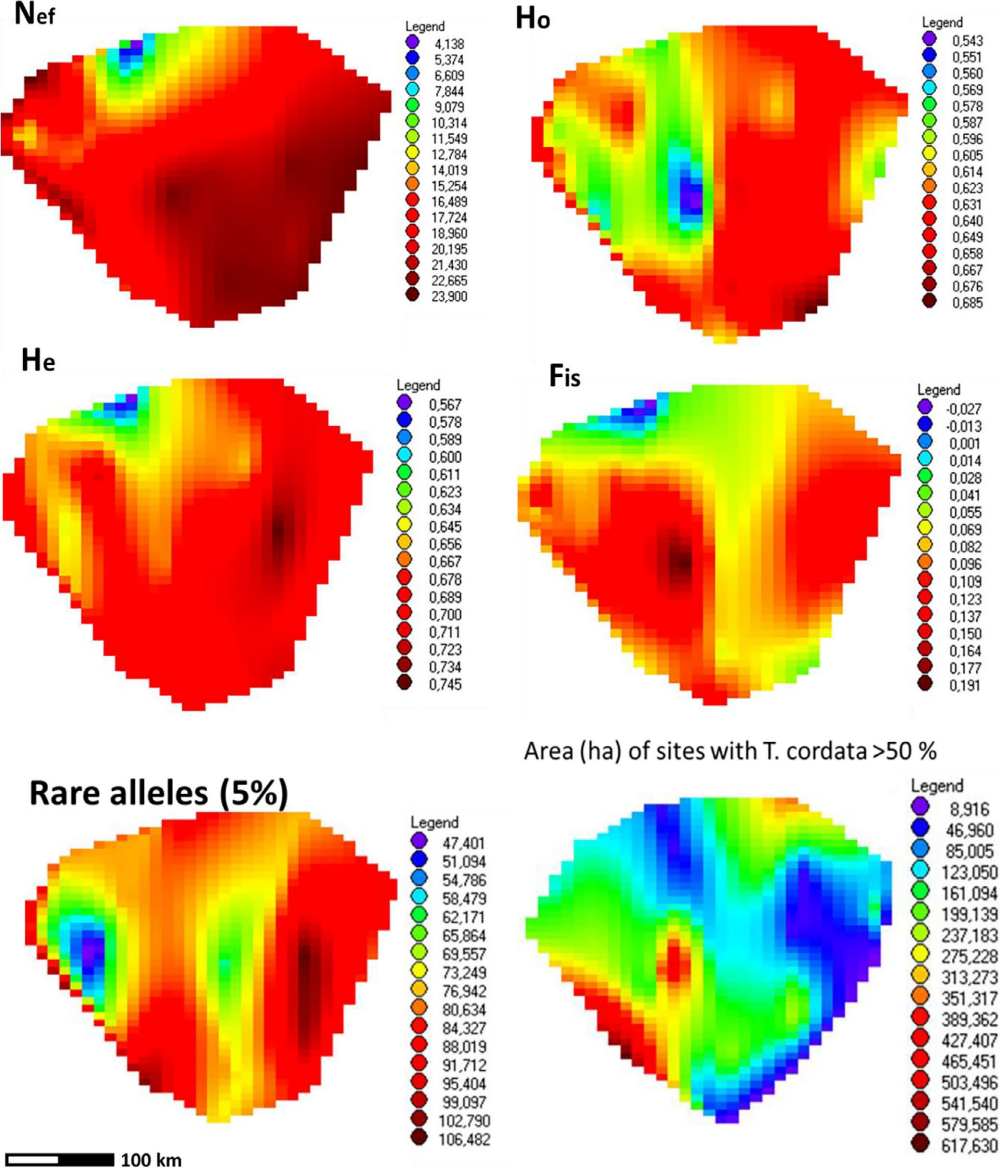
Interpolated surface plots of the within‐population genetic diversity parameters revealing the geographical patterns of genetic diversity of *Tilia cordata* in Lithuania. The shape of the plots is a simplified map of Lithuania. The actual population values are given in Table 3 and Figure S1. The lowest right surface plot illustrates geographical distribution *Tilia cordata*‐dominated sites (the statistics used on the map is the total area (in ha) of sites with >50% of *Tilia cordata* within a forest management unit of ca. 40 th. ha in size (forest inventory data from 2010)). N_ef_ – effective population size (the MIGRATE_N estimate), *H*_o_ and *H*_e_—observed and expected heterozygosity, and *F*_IS_—inbreeding coefficient. Rare alleles 5% is population number of alleles below the 5% frequency

Population fragmentation in *Tilia cordata* often leads to depleted diversity due to genetic drift, especially hazardous for entomophilous species with limited gene flow capacity (Erichsen et al., 2019; Lobo et al., 2018; Lowe et al., 2015). In addition to that, spreading from *Tilia* groups of likely low diversity in urban territories may further compromise the genetic stability of this newly expanding *Tilia cordata* populations. The results from the few available microsatellite‐based studies on *Tilia cordata* are diverse. Logan et al. (2015) found no marked reduction of genetic diversity of *Tilia cordata* in the UK, located not far from the margin of the range, whereas Lobo et al. (2018) reported possible genetic drift effects on *Tilia cordata* in Demark. Therefore, the situation with the genetic diversity of *Tilia cordata* may vary depending on the region. The above‐mentioned studies investigated *Tilia cordata* populations in rather fragmented forests surrounded by urbanized territories in western Europe. It would be interesting to obtain the genetic diversity and structure estimates for *Tilia cordata* from forested regions of Europe such as eastern Baltic forests.

The objective of our study was to assess the geographical distribution of the genetic diversity and the genetic structure of *Tilia cordata* populations in Lithuanian and discuss the main factors affecting the gene pool of *Tilia cordata* as well as its genetic potential for further spreading into mixed forest stands. For this purpose, we used a set of 14 genomic microsatellite markers to genotype a representative network of natural populations of *Tilia cordata* in Lithuania.

## 2 MATERIAL AND METHODS

### 2.1 Sampling sites

A total of 543 *Tilia cordata* individuals were sampled from 23 wild‐growing populations evenly covering the territory of Lithuania (Figure 1, Table 1). We carefully examined the chosen trees for *Tilia cordata* morphology to avoid unlikely but possible hybrids with *Tilia platyphyllos,* which is an exotic tree in Lithuania. We have not observed any potentially *T. platyphyllos* like individuals in the sampled stands (large hairy leaves with thick cuticula, angled fruits, multiple forking of stems due to frost damage). However, the northern PAPI population contained features less common elsewhere: pale bark with less expressed farrows, pronounced multiple forking of stems. The *Tilia cordata* sites were carefully chosen for natural origin and location by avoiding proximity to the urban areas, usually within forest tracts of various size. We used an electric drill to sample the sawdust from 20 to 25 adult overstory *Tilia cordata* trees per population. We selected the trees spaced at least 20 meters away from each other within a zigzag sampling path covering ca. 2–5 ha depending on the density of *Tilia cordata* at a site. In most of the stands we sampled, there were sites with more than 50% dominance of *Tilia cordata*.

**TABLE 1:** Location of the *Tilia cordata* populations sampled in Lithuania and the number of genotyped individuals

Region code	Population	Pop. Id.	Lat.	Long.	Alt.	Sample size
1A	Žagarė	ZAGA	56.29331700	23.21331400	88	20
1A	Papile	PAPI	56.20515583	22.91352556	84	24
1A	Šateikiai	SATE	56.02503405	21.67752476	100	25
1A	Varniai	VARN	55.70292792	22.50909472	195	24
1B	Juodkrantė	JUOD	55.54325700	21.11645000	19	26
1B	Rambynas	RAMB	55.05168500	22.10561000	52	22
1B	Pagramantis	PAGR	55.37100900	22.14515400	74	17
1B	Švėkšna	SVEK1	55.51747000	21.49529100	19	24
1B	Švėkšna	SVEK2	55.51749000	21.49529500	19	23
2A	Biržai	BIRZ	56.33503500	24.88834400	50	23
2A	Gegužine	PANE	55.80243267	24.51171700	62	26
2A	Kėdainiai	KEDA	55.29126106	23.87372242	56	23
2B	Veliuona	VELI	55.16233600	23.35795300	76	25
2B	Pažėrai	PAZE	54.82107300	23.70580900	78	25
2B	Degsne	DEGS	54.56889241	23.87390778	110	24
2B	Punia	PUNI	54.51995200	24.09517400	79	25
2B	Seirijai	SEIR	54.23238435	23.74611389	164	23
2B	Vyštytis	VYST	54.48111000	22.81771667	231	26
3	Dūkštas	DUKS	54.82652076	24.94313470	103	25
3	Anykščiai	KAVA	55.36664500	24.90653833	87	23
3	Kuktiškės	KUKT	55.37686700	25.82503100	215	22
3	Stelmužė	STEL	55.83252040	26.19646327	166	23
3	Pirčiupis	PIRC	54.42416500	24.98510542	139	25
					Total	543

Note

Region indicates climatic zones in Lithuania mainly affected by continentality and topography as shown in Figure 1. Region codes: 1—western (1A is coastal lowland and 1B is western highland), 2—midland lowland (2A and 2B for northern and southern parts), and 3—eastern highland.

### 2.2 DNA analysis

The DNA was extracted from silica gel‐dried wood sawdust collected by drilling with electric bore ca. 1 cm deep into the trunk (e.g., Verbylaitė et al., 2010, bark discarded, bore diameter 0.5 cm). A modified CTAB Doyle and Doyle (1990) DNA extraction method was used to extract DNA. For the genotyping, we used 14 genomic microsatellite DNA markers (Phuekvilai & Wolff, 2013, Table S1). The PCR amplification was carried on three multiplexes in a 15 µl reaction mix containing 7.5 µl of a PCR Master Mix, 3 µl of RNAse free water, 1.5 µl of Primer Mix, 1 µl of DNA, 1 µl of PVP 1 (%), and 1 µl of BSA 20 mg/ml (bovine serum albumin) (Applied Biosystems Thermo Cycler GeneAmp PCA System 9700) with following PCR thermocycling profile: initial denaturation step at 95°C for 15 min, followed by 25 cycles each of 94°C for 30 s, annealing temperature at 54°C for 1 min, 30 s, and extension at 72°C for 30 s, followed by the final extension step at 60°C for 30 min. Fragments were separated with the capillary electrophoresis on ABI PRISM™ 310 genetic analyzer. The alleles were scored on GENEMAPPER soft. ver. 4.1 (APPLIED BIOSYSTEMS). We constructed the allele scoring binset based on Phuekvilai and Wolff (2013, Table 1S) but later modified it for several loci according to our results (Table 2).

**TABLE 2:** Characteristics of the microsatellite loci from full dataset with 543 *Tilia cordata* individuals

Locus	Range, bp	Most frequent allele (freq.)	*N*_a_	*N*_e_	*H*_o_	*H*_e_	*F*_IS_	*G*_st_ (***)	*D*_est_ (***)	Null allele freq.
Tc6	122–146	132 (0.41)	13	4.4	0.78	0.78	−0.001	0.024	0.082	−0.006
Tc937	147–173	149 (0.54)	11	2.8	0.61	0.64	0.051	0.024	0.044	0.026
Tc920	219–245	233 (0.20)	14	8.3	0.82	0.88	0.068	0.041	0.240	0.033
Tc8^a^	140–166	140 (0.98)	4	1.0	0.01	0.08	0.932	0.152	0.014	0.176
Tc943	140–150	146 (0.58)	6	2.2	0.45	0.54	0.170	0.052	0.061	0.081
Tc4	215–243	231 (0.27)	15	7.0	0.65	0.86	0.243	0.028	0.154	0.119
Tc31	191 – 215	193 (0.61)	12	2.4	0.39	0.58	0.334	0.043	0.058	0.171
Tc927	141 –181	141 (0.85)	9	1.4	0.25	0.27	0.069	0.025	0.010	0.029
Tc11^a^	131 – 151	131 (0.65)	4	1.8	0.29	0.46	0.356	0.044	0.038	0.161
Tc915	143 – 181	151 (0.32)	20	5.7	0.80	0.83	0.030	0.031	0.133	0.013
Tc963	235 – 301	273 (0.12)	30	14.1	0.75	0.93	0.197	0.024	0.254	0.099
Tc951	151 – 163	159 (0.58)	7	2.5	0.46	0.59	0.219	0.046	0.066	0.102
Tc5	135 – 175	147 (0.34)	19	4.3	0.76	0.77	0.012	0.021	0.068	0.003
Tc7	234 – 266	244 (0.30)	13	5.8	0.79	0.83	0.041	0.032	0.141	0.021
			178^b^					0.034^c^	0.060^c^	

Note

The frequency of the most frequent allele is given in the brackets. *N*_a_ is number of different alleles. *N*_e_ is effective allele number. *H*_o_ and *H*_e_ are observed and expected heterozygosity. *F*_IS_ is the FSTAT inbreeding coefficient. *G*_st_ and *D*_est_ are frequency‐based differentiation indexes among the 23 populations (*** indicates that all were significant at 0.001 level with 9,999 GeneAlex permutations). Null allele frequency by Oosterhout method. For all the loci, the microsatellite repeat motive is 2 bp.

^a^ The excluded loci from the population genetic structure and diversity analysis.

^b^ Sum of different alleles.

^c^ Multilocus estimate from the GeneAlex tests.

### 2.3 Data analysis

We assessed the frequency of null alleles for each locus with all populations pooled with MICROCHECKER soft. ver. 2.2.3 (Van Oosterhout et al., 2006). Occurrence of clones and the standard genetic diversity parameters along with the Mantel test statistics for isolation by distance were calculated with GENALEX soft. ver. 6.5 (Peakall & Smouse, 2006). The rarefied allelic richness (Ar, base 17 chosen for the lowest sample size in population PAGR, Table 1) and the inbreeding coefficient (Fis along with the significance of its deviation from 0) were calculated with FSTAT soft. ver. 2.9.3.2 (Goudet, 1995). We tested the significance of differences between the regions in main genetic diversity parameters by using the FSTAT among group significance tests based on 1,000 permutations.

The effective population size (*N*_ef_) was calculated for each population and region based on maximum‐likelihood method (Hastings–Metropolis Markov chain Monte Carlo algorithm) and coalescent theory using MIGRATE_N software (Beerli, 2009). This program returns the theta (ϴ) value, which was used to calculate the effective population size *N*_ef_ = ϴ/μ, where *μ* is assumed mean microsatellite mutation rate per generation of 4 × 10^−3^ (Boys et al., 2005; Pandey & Rajora, 2012). We also use MIGRATE_N to calculate the number of inward and outward migrants per generation at the population and region levels, to obtain an estimate of gene flow patterns in *Tilia cordata* in Lithuania.

We run the Wilcoxon rank test for heterozygosity excess and the mode shift test to screen for bottleneck effects on the region level with software BOTTLENECK 1.2.02 (Cornuet & Luikart, 1997). The Wilcoxon rank test (Luikart, 1997) assumes that in a population after a recent bottleneck, the expected heterozygosity is decreasing more slowly than the allele dropout. Therefore, the expected heterozygosity (*H*_e_) observed in the data (or homogeneity of allelic frequencies) is higher than H_e_ expected under mutation‐drift equilibrium (Garza & Williamson, 2001). The software tests for significant number of loci that have *H*_e_ excess. This assumption suites better for the loci under the IAM (infinite allele model) than SMM (stepwise mutation model) (Cornuet & Luikart, 1997). However, we tested all three mutation models: SMM, IAM, and two‐phase mutation (TPM). In the Wilcoxon rank test statistics, we checked the one‐tail *p*‐values for *H*_e_ excess and examined the homogeneity of the one‐tail p‐values for the *H*_e_ excess and deficit (because in a population at mutation‐drift equilibrium; there is approximately an equal probability that a locus shows H_e_ excess or deficit). The mode shift tests for deviations from so‐called L shape distribution of allele frequencies usually indicate recent bottlenecks. This test assumes that the bottleneck‐free populations possess high number of low‐frequency alleles which follow an L shape appearance of the allele frequency histogram (Luikart, 1997).

The interpolated surface plots of genetic diversity parameters were obtained by calculating the matrix of interpolated values with the MS EXCEL function =@Interp2d and then drawing the matrix rainbow plot by the id of the POPTOOLS EXCEL add‐in.

The population differentiation was tested by calculating (a) the frequency‐based differentiation indexes G_st_ (an analogue of F_st_ adjusted to variable sample size) and D_est_ (Jost, 2008) with GENALEX soft. ver. 6.5 and (b) running an AMOVA to partition the molecular variance among regions, populations within regions, and within populations with Arlequin soft. ver. 3.5.1.3 (Exoffier & Lischer, 2010).

We used the Bayesian clustering approach implemented in STRUCTURE soft. ver. 2.2.3 (Pritchard et al., 2000) to investigate the population genetic structure of *Tilia cordata* in Lithuania. We set the burn‐in period length for posterior distribution to 10^5^ and the number of MCMC iterations to 10^5^, the K range from 1 to 10, each replicated 10 times. We used the correlated allele frequency model, no admixture, and the LOCPRIOR option for the three regions indicated in Table 1 and Figure 1. The most likely number of genetic clusters K was identified based on the deltaK value (Evanno et al., 2005) with STRUCTURE_HARVESTER WEB soft. (Earl & von Holdt, 2012). In genetically heterogeneous material especially under presence of botanical hierarchy (e.g., hybridization or transfer effects), the optimal number of genetic groups may be underestimated (e.g., Wang, 2017). Therefore, we presented the genetic structures for a range of *K* values from 2 to 6. We did not consider deploying higher than *K* = 6 groups because the individual Bayesian assignment started to “decay” so that for a significant number of individuals the membership proportions become similar between the *K* groups tested. Within each run of *K* from to 10 clusters, the most appropriate replication out of 10 was identified by (a) calculating for each individual the maximum likelihood for belonging to a single cluster out of N clusters and (b) averaging these maximum likelihoods over all individuals for each single run and (c) selecting a single run out of 10 replicated runs with the highest mean likelihood. In this way, we obtained the structure, which assigned individuals to a single cluster with the highest likelihoods.

We used Monmonier's algorithm allowing for establishing barriers along a significant shift in the allele frequency within a landscape implemented in soft. BARRIER ver. 2.2 (Manni et al., 2004). The program (a) creates a Delaunay triangulation plot between the sampled populations, (b) calculates genetic distances (Nei et al., 1983) associated with each edge in the plot, and (c) creates growing barriers along the largest genetic distances on the plot; the barriers are ranked based on the magnitude of the differentiation. The program also allows for a significance test of the barriers by analyzing the bootstrapped distance matrices and displaying the number of events returning a given barrier. We calculated 100 bootstrapped Nei et al. (1983) genetic distance matrixes among the 23 populations with MICROSATELLITE ANALYSER soft. ver. 4.05 (Dieringer & Schlotterer, 2003).

Finally, we used the ALLELES IN SPACE soft. ver. 1.0 (Miller, 2005) to obtain a surface plot of the interpolated Nei et al. (1983) genetic distances among *Tilia cordata* populations in Lithuania.

We also screened for possible natural hybridization events between *T. cordata* and *T. platyphyllos* by examining the geographical distribution of the less common alleles at the two loci Tc8 and Tc927. These loci were reported to discriminate well between these two *Tilia* species (Logan et al., 2015). The authors reported that the locus Tc8 was monomorphic in the UK with *T. cordata* material (the 96% frequent 140 bp allele in our material) but returned 8 alleles in *T. platyphyllos* and the locus Tc927 few alleles in *T. cordata* and 15 alleles in *T. platyphyllos*.

## 3 RESULTS

### 3.1 Loci statistics

We detected no clones among the 543 trees of *Tilia cordata* in our material. All the loci consistently contained two alleles per individual, indicating no polyploidy in *Tilia cordata*. The set of 14 loci generated a total of 178 different alleles (average 12.7 alleles per locus). For most loci, the expected heterozygosity (*H*_e_) values varied at about 0.8 indicating highly polymorphic material (Table 2). The *H*_o_ values ranged from 0.25 to 0.78, indicating stronger deviations from random mating than in the wind‐pollinated species such as Scots pine. The loci TC8 and TC11 were least informative, monomorphic in most of the populations, possessed high frequency of null alleles (0.17 and 0.16, respectively), and therefore were excluded from further analysis of genetic structure and diversity (Table 2). All the other loci had null allele frequencies below 0.1 except locus TC31 (freq. null = 0.17, Table 2). The locus TC31 was highly informative and the inbreeding coefficient at this locus varied markedly among populations (not shown), implying that null alleles at this frequency of 0.17 do not markedly deviate the F_is_ values.

### 3.2 Within‐population genetic diversity

Within‐population genetic diversity varied markedly among the populations. The allelic diversity (allelic richness, *A*_r_ and expected heterozygosity, *H*_e_) in the western region 1 was significantly lower than in the remaining regions (Figure 2, Table 3, Figure S1). In western Lithuania, 5 of 8 populations possessed the lowest ranks of *H*_e_ from 0.56 to 0.65, including the two adjacent northeastern populations of PAPI and ZAGA (Table 3, Figure 2, Figure S1). The PAPI and ZAGA populations also showed a high degree of differentiation from the rest in his region (subsection below and Figure 4). There were no significant regional differences in the observed heterozygosity (*H*_o_) and inbreeding coefficient (*F*_IS_) (Table 3). However, we observed a tendency of lower H_o_ values and higher *F*_IS_ values in coastal lowland, even though Tilia cordata is most common there (region 1B, the former Eastern Prussia with 3 out of 4 populations with *H*_o_ < 0.6 Table 3 and Figure 2). Elevated *F*_IS_ values were also observed in the eastern highland, where climate is cooler, soils are poor and dry but *Tilia* is least common (region 3, Figure 2).

**TABLE 3:** Within‐population genetic diversity parameters

Pop	*N*_obs_	*N*_a_	*N*_e_	*H*_o_	*H*_e_	A_r17_	*F*_IS_	p*F*_IS_	*N*_ef_	Rare5%
1‐ Western (western highland and coastal)										
ZAGA	20	6.67	3.70	0.61	0.64	6.19	0.035	0.1696	10.0	88
PAPI	24	4.67	3.03	0.58	0.56	4.34	−0.039	0.8772	3.0	78
SATE	25	7.50	4.04	0.63	0.65	6.54	0.040	0.1074	24.6	79
VARN	24	7.75	4.34	0.64	0.72	6.77	0.106	0.0004	15.2	78
JUOD	26	9.00	4.82	0.65	0.71	7.56	0.087	0.0016	22.0	101
RAMB	22	6.58	3.60	0.56	0.65	5.92	0.136	0.0002	23.4	57
PAGR	17	6.50	3.64	0.58	0.64	6.50	0.088	0.0205	15.3	42
SVEK1	24	7.08	4.03	0.65	0.70	6.33	0.069	0.0178	14.8	64
SVEK2	23	5.58	3.56	0.58	0.68	5.10	0.151	0.0002	10.5	63
Mean		6.8	3.86	0.61^A^	0.66^A^	6.14^A^	0.058^A^	–	15.4	72.2
*SE*		0.31	0.22	0.02	0.02	0.17	0.021	–	2.4	4.8
2‐ Midland lowland										
BIRZ	23	8.08	4.28	0.66	0.71	7.08	0.076	0.0078	17.7	77
PANE	26	6.75	3.86	0.61	0.65	5.88	0.070	0.0194	17.5	74
KEDA	23	7.33	4.25	0.66	0.71	6.62	0.065	0.0254	22.3	60
VELI	25	7.17	4.07	0.53	0.66	6.39	0.203	0.0002	23.4	82
PAZE	25	8.58	4.27	0.64	0.71	7.30	0.095	0.0007	22.0	79
DEGS	24	7.67	4.61	0.65	0.70	6.89	0.077	0.0062	23.4	73
PUNI	25	7.42	4.26	0.66	0.70	6.60	0.060	0.0243	22.5	79
SEIR	23	8.00	4.31	0.61	0.69	7.06	0.126	0.0002	21.7	74
Mean		7.7	4.22	0.63^A^	0.69^B^	6.73^B^	0.076^A^	–	20.7	78.1
*SE*		0.33	0.22	0.02	0.02	0.17	0.019	–	0.9	4.0
3‐ Eastern highland										
VIST	26	8.25	4.06	0.66	0.71	6.85	0.071	0.0098	16.0	105
DUKS	25	7.33	3.95	0.63	0.70	6.42	0.103	0.0013	21.8	104
KAVA	23	8.00	4.82	0.65	0.75	7.16	0.126	0.0002	23.3	107
KUKT	22	7.75	4.15	0.58	0.68	6.92	0.146	0.0002	23.7	82
PIRC	23	8.08	4.65	0.69	0.72	7.02	0.036	0.1409	23.4	88
STEL	25	7.92	4.35	0.63	0.70	6.79	0.102	0.0011	22.1	90
Mean		7.8	4.38	0.64^A^	0.71^B^	6.86^B^	0.085^A^	–	22.9	94.2
*SE*		0.44	0.32	0.03	0.02	0.21	0.021	–	0.4	4.8

Note

*N*_obs_ is the sample size; *N*_a_ is number of different alleles. *N*_e_ is effective allele number, *H*_o_ and *H*_e_ are observed and expected heterozygosity, A_r17_ is rarified allelic richness with the base of 17 individuals, F_is_ is the FSTAT inbreeding coefficient and its significance (p*F*_ST_, 5,000 permutations), *N*_ef_ is effective population size (coalescence in MIGRATE_N), and Rare5% is number of rare alleles below the 5% frequency. The regional means of the genetic diversity parameters with the same latter are not significantly different according to the pairwise FSTAT significant tests between region means at 0.05 significance level of the two‐sided p‐value based on the 1,000 permutation. *SE* is standard error calculated from the population/locus mean values (except *N*_ef_ from population mean values).

Geographical distribution of the effective population size N_ef_ followed that of H_e_ with the lowest values in western highland and highest—in eastern highland (Figure 2, Table 3, Figure S1). Finally, rare allele numbers peaked in the eastern highland (region 3) and dropped to the lowest ranks in coastal lowland (region 1B, Table 3, Figure 2).

The Wilcoxon bottleneck test following the SMM and TPM models returned no significant heterozygosity excess with the *p*‐values ranging from .8 to .9 for all five regions. The mode shift test showed no deviations from the normal L‐shaped distribution for all 5 regions. However, the Wilcoxon test following the IAM model detected markedly lower one‐tail p‐values for the H_e_ excess in the regions 1B (coastal lowland, *p* =.08) and 3 (eastern highland, *p* =.02), while for the remaining regions, the p‐values were well above the significance threshold and ranged from 0.2 to 0.4 (Figure 5). For all the mutation models and regions, the *p*‐values for *H*_e_ excess and deficit differed markedly, pointing at deviation from the mutation‐drift equilibrium.

### 3.3 Genetic differentiation and structure

The frequency‐based differentiation tests at all loci retuned significant differentiation values between the populations of *Tilia cordata* in Lithuania (the multilocus *D*_est_ = 0.02 with *p* =.001 and *G*_st_ = 0.034 with *p* =.001). The set of loci that returned the strongest *D*_est_‐based differentiation was Tc920 (*D*_est_ = 0.24), Tc4 (0.15), Tc915 (0.13), Tc963 (0.25), Tc7 (0.14). These five loci also were the most polymorphic with highest ranking N_a_ and H_e_ values (Table 2). The AMOVA revealed weak but significant differentiation among the three main regions (1, 2, and 3) (variance component 0.21%, the *p*‐value = .0431), strong differentiation among populations within region (3.53%, *p*‐value = .00001), and the remaining 96.26% between individuals within populations (Table S2).

The Mantel test showed weak but significant linear correlation between the genetic and geographical distances (*R*_xy_ = 0.047, permuted *p*‐value = .001), indicating some isolation by distance and substructuring among *Tilia cordata* populations in Lithuania.

**FIGURE 3:**
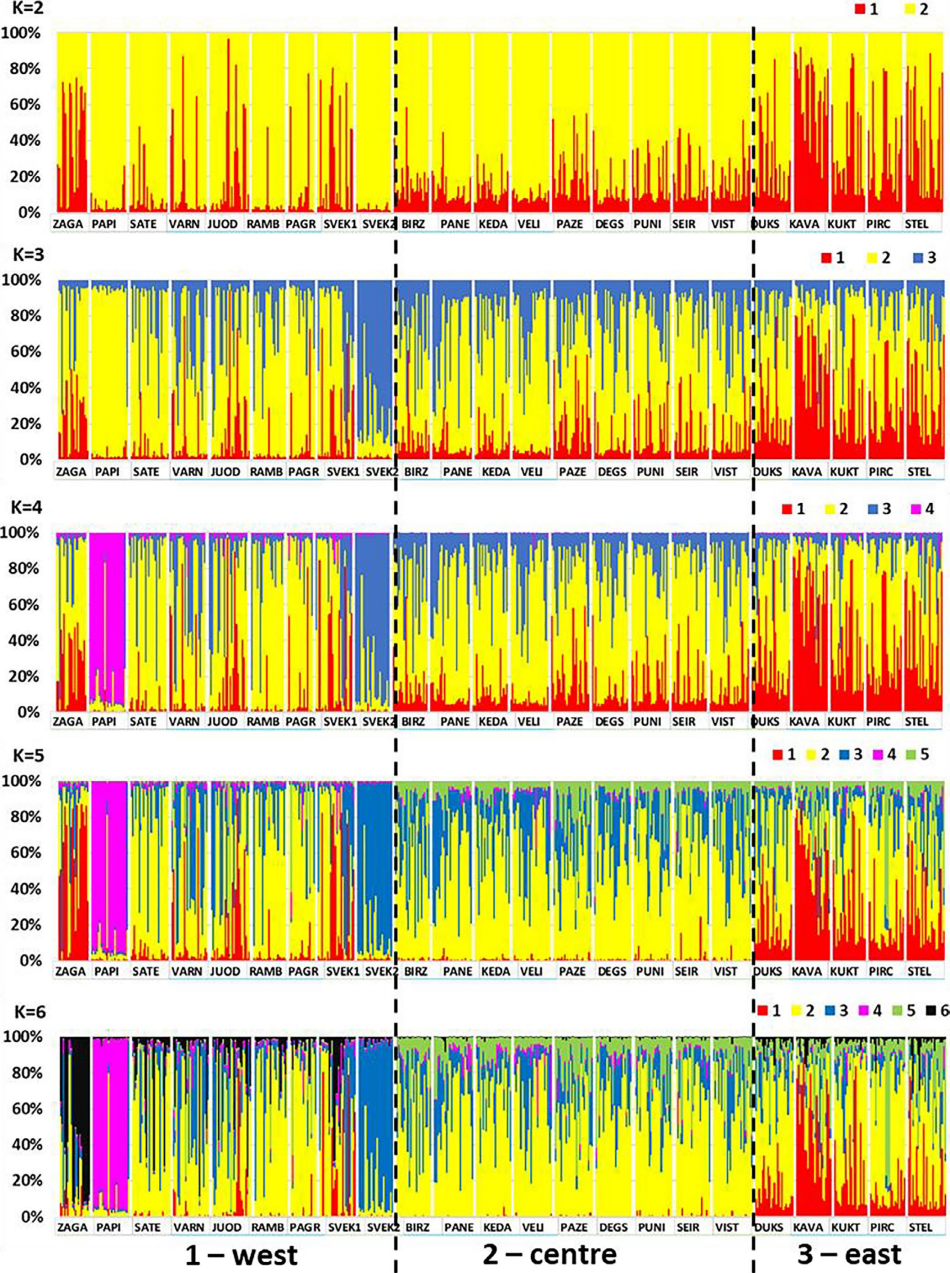
Membership proportions of *Tilia cordata* individuals in *K* = 3–6 clusters inferred by the STRUCTURE Bayesian clustering analysis. Population ids. are given on the x‐axis. The populations are arranged by the three large regions separated by dashed vertical lines. The highest deltaK value was obtained for the *K* = 4 cluster structure

Evanno's deltaK method suggested *K* = 4 as the optimal number of the Bayesian clusters based on the 10 replicated STRUCTURE runs for each *K* from 1 to 10 (Figure S2). However, by considering the observation that the STRUCTURE_HARVESTER algorithm tends to underestimate deltaK in a genetically heterogeneous material (Hintsteiner et al., 2018) and seeking for a finer structure, we considered the population structure under a range of K values from 2 to 6 (Figure 3). At *K* = 2, there was a clear differentiation of the eastern populations (Figure 3). At *K* = 3, the SVEK2 population from the former Eastern Prussia emerges, surprisingly differentiated from the adjacent SVEK1 (ca. 2 km apart from each other). Here, we need to note the morphotype difference between SVEK1 and SVEK2 (not scored), which was the reason for sampling these two geographically adjacent stands. In SVEK2, straight and single‐stemmed trees dominated, while SVEK1 contained large number of curvy trees with multiple forking. Both the stands had no signs of coppicing. At *K* = 4 to 5, the PAPI population in the northeast emerged with no associates at all and the homogeneity of the midland lowland populations starts to be more pronounced (Figure 3). Finally, at *K* = 6, a cluster separating the western populations emerges with the highest frequency for the northeastern ZAGA population. At the *K* > 6 values, no new geographical interpretable groups emerged, and the structure becomes less stable as the single cluster membership proportions started to “decay”, that is, be equally shared within the individuals (not shown). Therefore, we consider the *K* = 6 structure interpretable as optimum in our material.

**FIGURE 4:**
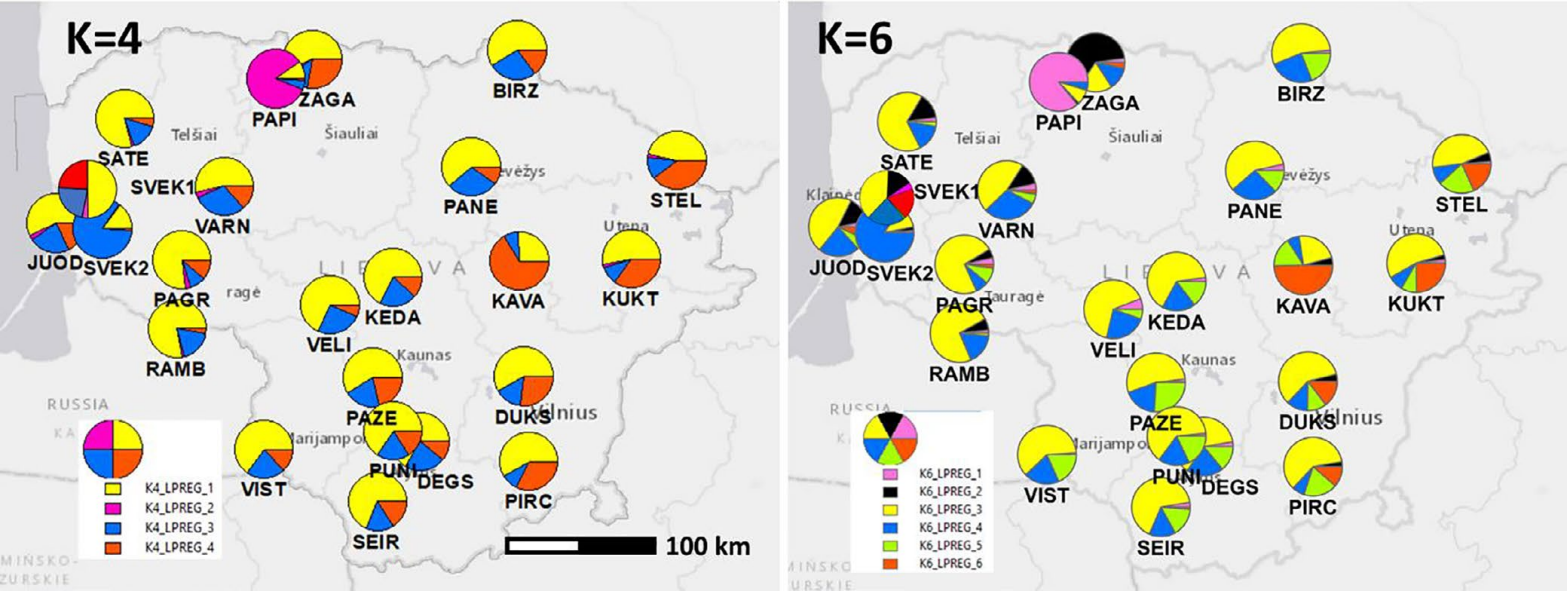
Geographical distribution of the membership proportions of 543 *Tilia cordata* individuals from 23 populations into the four (left) and six (right) clusters as inferred by the Bayesian clustering analysis with the STRUCTURE software

Geographical distribution of the membership proportions into the *K* = 6 clusters revealed the following structure (Figure 4): (a) high genetic heterogeneity of populations in western region 1, where several rare clusters occurred at high frequency and several geographically close populations were assigned to different genetic clusters (e.g., PAPI‐ZAGA, and SVEK1‐SVEK2 in Figure 4); (b) a rather genetically homogeneous group of populations (region 2), free of the genetic outliers as in western and eastern Lithuania, and (c) the most genetically differentiated population group located in the eastern highland sharing elsewhere rare cluster in relatively higher proportions (e.g., cluster id. 6 reaching 50% in the KAVA population in Figure 4).

The additional STRUCTURE runs to screen for a finer structure by analyzing a subset of populations revealed (a) a subdivision of the western region 1 into northern 1A and southern 1B, in this way separating the former Eastern Prussian territory from the rest (b) slight differentiation of the two northern populations of PAPI and ZAGA from the rest in the midland highland (Figure S5).

The Monmonier's barriers along marked shifts in allele frequencies were not as helpful in finding geographical consistent genetic structures (Figure S3). The barriers were drawn around most of the populations, especially in the eastern and western regions (Figure S3). Therefore, a more meaningful interpretation of this analysis is identifying areas where the barriers are not identified given maximum reasonable occasions for identifying the barriers. Consequently, under the settings to identify 10 the most significant barriers given 100 bootstrapped matrices, the midland lowland was the only large‐scale barrier‐free region (Figure S3).

The surface plot of the interpolated Nei et al. (1983) genetic distances basically differentiated eastern the eastern highland from the remaining regions, which confirms the structure revealed by the Bayesian clustering (Figure S3).

As regards possible hybridization between *T*. *cordata* and *T. platyphyllos*, we examined the putative *T*. *platyphyllos* marker allele frequencies for the loci Tc8 and Tc927. For the locus Tc8 with the most common 140 and 146 bp alleles at 99% frequency (Table 2), T. platyphyllos 156‐bp and 166‐bp marker alleles were found in single individual in JUOD population out of all 543 trees. For locus Tc297, the highest and lowest concentration of the four rarest alleles out of the total of nine was in central and northeastern Lithuania, respectively (Figure S4).

## 4 DISCUSSION

### 4.1 Genetic features of *Tilia cordata* in Lithuania

Our study revealed that despite the speculated threats for fragmentation and genetic drift under a biologically limited gene flow capacity, *Tilia cordata* retain high genetic diversity in Lithuania (in agreement with Erichsen et al., 2019; Logan et al., 2015). However, the range of genetic diversity *H*_e_ values obtained in our study (*H*_e_ = 0.66–0.71) exceeded those found by Logan et al. (2019) for *Tilia cordata* in Poland (*H*_e_ = 0.53 – 0.58), Russia (*H*_e_ = 0.56–0.59), and Austria (*H*_e_ = 0.61) as well as average *H*_e_ values in the UK (*H*_e_ = 6.3, Barker, 2017), in central and range‐edge across Europe 0.56–0.57, respectively (Logan et al., 2019), average 0.62 across Denmark (Erichsen et al., 2019). The genetic diversity levels were even comparable with wind‐pollinated species such as Scots pine in Lithuania (Danusevičius et al., 2016). The inbreeding estimates in our study were lower than in the Danish material of *Tilia cordata*, where more asexual reproduction exists (Erichsen et al., 2019).

To preserve such high levels of genetic diversity, an evolving network of natural populations is required (De Jaegere et al., 2016). Therefore, it is likely that autochthonous gene pool of *Tilia cordata* must have been maintained by synergy of gene flow, natural selection, and genetic drift in the network of forest tracts in Lithuania. Assuming a similar landscape management history and ecoclimatic conditions in the Baltic region and southwestern parts of European Russia, our results may be generalized onto a broader geographical scale. In 1700s and 1800s, the forest cover of Lithuania was 60% and 50%, respectively, followed by a gradual reduction to ca. 20% due to forest feelings during WW2 (Kairiūkštis, 2003). Then, presumably, *Tilia cordata* experienced further reduction in population size surviving in the deep of large woodlands. It is likely that these ancient woodlands especially on moist and rich soils in the lowlands were of sufficient ecological capacity to sustain a sound network of *Tilia cordata* populations. In contrast, rural areas of the UK and Denmark are more urbanized likely leading to stronger imprints of low diversity urban escapes on natural gene pool of *Tilia cordata*.

Another explanation for the relatively higher genetic diversity of Lithuanian *Tilia cordata* populations could be lower rates of asexual reproduction, which is sensitive to genetic drift effects (Balloux et al., 2003). The fact that we did not detect any clones in the 543 sampled trees indicates that the 20‐m sampling distance among trees is safe to avoid clonal samples of *Tilia cordata*. Most of the trees had no morphological features for clonal sprouting (bending at the base or multicormic trees). Asexual reproduction in *Tilia* is expected to increase when moving northwards due to harsher climate for sexual reproduction (De Jaegere et al., 2016). With warming climate, however, more sexual reproduction is expected in the northern Tilia populations as discussed by Logan et al. (2019) and supported in our study by low frequency of clonal morphotypes (multiple stems bending at the base of the trunk) and absence of identical microsatellite genotypes.

Based on the Tc8 marker (Logan et al., 2015), the hybridization between *T. cordata* and *T. platyphyllos* is very low (detected at low rare in a single coastal population of JUOD). T. *platyphyllos* does not occur naturally in the forests and is present in urban parks of Lithuania. JUOD population is in the coastal spit, presently a national park, formerly a famous coastal resort of Eastern Prussia, where we personally found small, planted groups of T. platyphyllos some 2–3 km away from the sampling site. The single case tracked by us indicates that spontaneous hybridization or escapes are possible and likely to intensify in the future. To confirm that, Semerikova et al. (2020) in a cpSSR study observed putative Tilia cordata x platyphyllos hybrid near St. Petersburg and interpreted it as an escape from famous royal parks. With another putative hybrid marker for the *Tilia* sp. Tc927, the results are less clear. Most populations contained ca. 3% frequency of rare alleles, except a few (Figure S4), but these were natural populations with no direct evidence of mixture with urban gene pools.

In agreement with the earlier studies on Tilia sp. with the SSR markers (Barker, 2017; Erichsen et al., 2019; Lobo et al., 2018; Logan et al., 2015), we did not detect more than 2 alleles at all loci, confirming the diploidy of *Tilia cordata*. In our study, the observed heterozygosity estimates are not markedly affected by null alleles as shown by the MICROCHECKER tests and the high variation in the singe locus *H*_o_ and *F*_IS_ values among the populations.

### 4.2 Within‐population diversity is geographically variable in Lithuania

We clearly observed low values of allelic diversity and tendency of elevated inbreeding in western Lithuania, especially in region 1B with the possible bottleneck effects in the past. There could be two major causes for that (a) fragmentation drift in natural gene pools and (b) aftereffects of spreading from urban sources of low diversity (Hemery et al., 2010). Tilia cordata is not rare in region 1B (Figure 2), which makes the earlier of the two factors less likely. As regards the later, the urban territory managers hardly took the effort of going into the deep of the gloomy forests for *Tilia cordata* seeds. It is likely that the seeds for urban *Tilia cordata* plantings were collected from limited number of trees in old manor parks. If *Tilia cordata* from such sources escaped into the wild, low allelic diversity and elevated inbreeding are expected in the progeny. This escape may be fastened by human mediation such as transporting wasted autumn leaves (and seeds) of park trees into nearby forests. Therefore, artificial establishments may constitute a significant spreading source of *Tilia cordata* back into the forests and leave a notable signature into the ancient autochthonous gene pools. This observation is confirmed by relatively higher genetic heterogeneity of Tilia cordata in region 1 (Figure 4).

For post‐WW2 urban plantations, the original sources of *Tilia cordata* are unknown, but likely to be old parks and seldom autochthonous forest sources (Tauras, 1989). Importantly, the old manor park designers over centuries often used nonlocal collections and botanical gardens as the seed sources to surprise their customers (Rosłon‐Szeryńska et al., 2020). Therefore, all this urban source spreading of *Tilia cordata* is likely to contain nonlocal sources of unknown adaptedness in the wild.

In Lithuania, *Tilia cordata* has its highest frequency in the western part (Figure 2). How could it be that these most *Tilia‐*rich areas show lowest genetic diversity? A possible answer is significant effects of the urban gene pools, and the urban escapes may not be recent. Western Lithuania has a high concentration of old manor parks (Tauras, 1989). Good candidates for such urban escapes are the two adjacent PAPI and ZAGA populations (northwest): lowest allelic diversity and heterozygosity values (Table 3, Figure S1), or the SVEK2 population in the former Eastern Prussia with the distinct morphology and the genetic identity outstanding from the adjacent SVEK1 and the remaining populations in this region (Figure 4). Use of nonlocal material for forestry was not uncommon in the former Eastern Prussia (Kembrytė et al., 2021).

**FIGURE 5:**
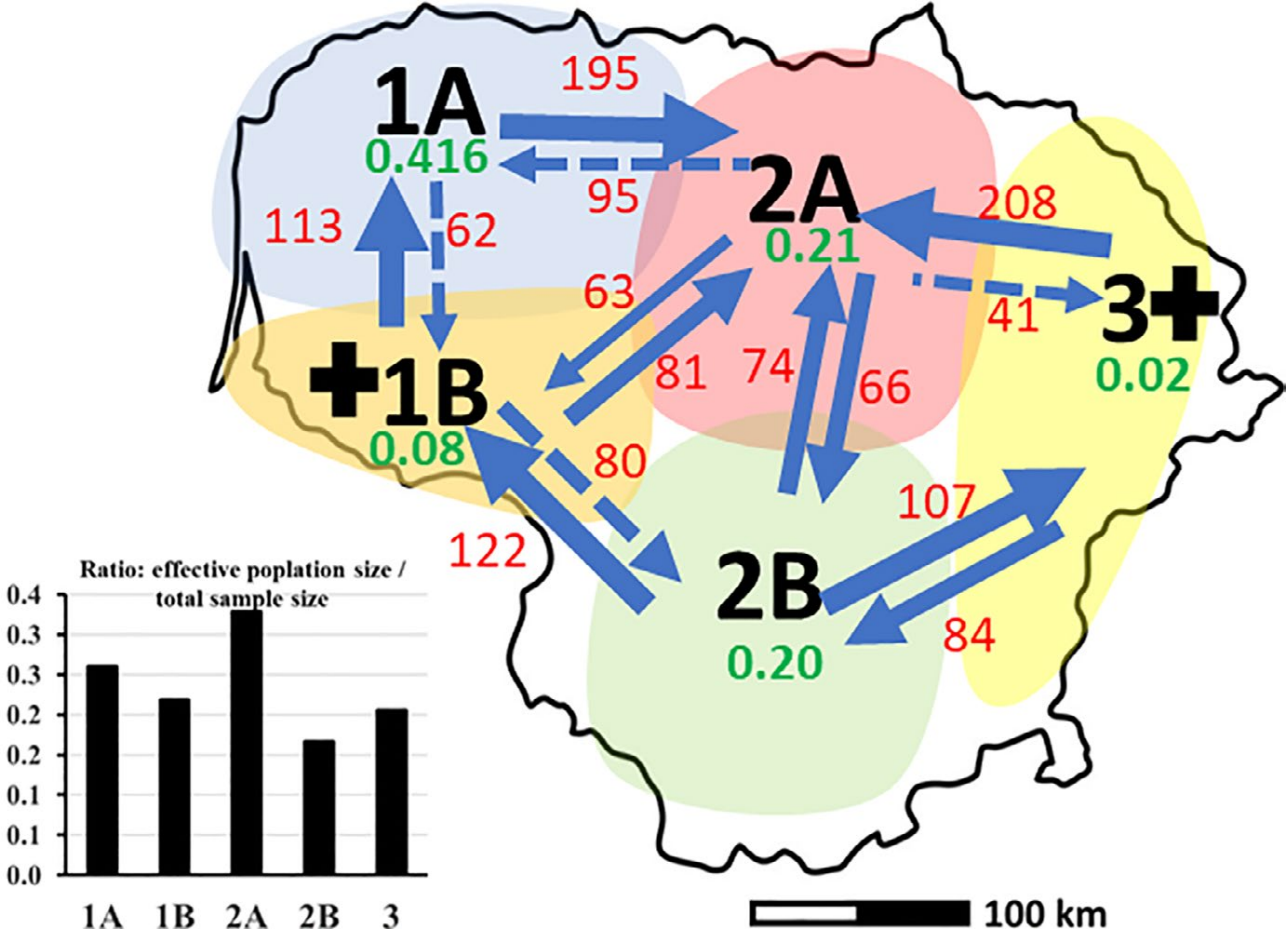
Patterns of gene flow and tests for bottleneck effects in *Tilia cordata* populations. The p‐values for the expected heterozygosity excess from the one‐tail WILCOXON test for the bottleneck effect are given below the region codes in green (the black cross at the region code indicates bottleneck effects in the past). Estimates of gene flow (*N*_m_, individuals per generation) and the effective population size (*N*_ef_ the bar chart, lower left) were estimated with the MIGRATE_N soft. among the regions. The inward and outward *N*_m_ estimates are given at the arrows in red numbers (smaller dashed arrows signify much weaker bilateral change). The regional characteristics are as follows: 1A—western highland (sample size *N*_census_ = 93, effective population size *N*_ef_ = 24.2), 1B—coastal lowland, mainly former Eastern Prussia (*N*_census_ = 112, *N*_ef_ = 24.5), 2A—northern part of midland lowland (*N*_census_ = 72, *N*_ef_ = 23.7), 2B—southern part of the midland lowland (*N*_census_ = 148, *N*_ef_ = 24.6), 3—eastern highland (*N*_census_ = 118, *N*_ef_ = 24.3)

In the eastern Lithuanian highlands (region 3), the situation with genetic diversity was different—high allelic diversity but elevated inbreeding and bottleneck effects detected (Table 3, Figures 2 and 5). Such situation with deviations from random mating may occur when the intramating groups differentiate within the natural gene pools. This group differentiation leads to the Wahlund effect (lower H_o_ than H_e_ values within a population caused by substructuring) that could be caused can be strong fragmentation due to reduction of the effective population size under higher selective pressure in this coolest region in the country (as indicated by the significant bottleneck effect). There also is a relatively higher urbanization pressure in eastern region 3 (Figure S5) that may lead to formation of internally mating groups we well. Another possible reason could be more recent natural spreading of the western Russian *Tilia cordata* gene pools because of warming climate (supported by a higher rare allele frequency in the eastern region 3, Figure 2; McLachlan et al., 2005). In the long run, these eastern immigrants may help to recover after the bottleneck effect.

Moist and rich sites such as the midland lowland (region 2A) create favorable environment for *Tilia cordata* (e.g., Barker, 2017) allowing for larger and less fragmented populations in midland lowland (Figure S5). This may explain relatively lower inbreeding values for *Tilia cordata* populations in midland lowland.

### 4.3 Genetic structure is associated with geography in Lithuania

Our results on genetic structure of *Tilia cordata* in Lithuania contrast with Erichsen et al. (2019) study where no clear population structuring was found for *Tilia cordata* in Demark with the set of SSR markers. Based on the findings discussed above, this genetic structure is likely to be shaped by the balance between drift caused fragmentation and gene flow as well as local effects of urban escapes (Lobo et al., 2018). There also could be signs of adaptation to poor soils and relatively higher frost tolerance in the coldest region in Lithuania—the northeastern highland.

Theoretically, insect pollination implies weak gene flow among populations (e.g., Lowe et al., 2015). Hence, the gene flow rates obtained by the coalesce algorithms in our study may also reflect seed migration vectors mediated by wind, water, and birds (Calladine & Morrison, 2010; De Jaegere et al., 2016). The first observation on gene flow patterns is free mutually equal genetic exchange among the midland lowland regions 2A and 2B (confirmed by the BARRIER structure analysis, Figure 2 and Figure S3). An explanation for this could be seed transport via a riparian network of the largest river in Lithuania–Nemunas and its affluent Nevezis. A relatively stronger gene flow from the flanking highlands (regions 1 and 3) downwards into the midland lowland (region 2) also supports the riparian gene flow hypothesis (Figure 5). A uniform adaptive environment of the midland lowland could contribute to homogeneous genetic structure in this region as well. The second observation on gene flow is on the outward flow from the bottlenecked regions being stronger than the inward flow (Figure 5). This may indicate that spreading of *Tilia cordata* is more successful on the optimum sites such as the most and rich soils of midland lowland (region 2) than elsewhere. In our material, there also is a tendency for a stronger northward than southward directed gene flow (Figure 5), supporting the observations on advance of *Tilia* northwards (Logan et al., 2019).

As regards the genetic structure of *Tilia cordata* in Lithuania, we found three regions showing a genetic structuring as follows: (1) high genetic heterogeneity of the populations in western Lithuania, (2) genetically homogeneous population composition in the midland lowland, and (3) unique genetic group present in high frequency in the northeastern highland. We interpret the high genetic heterogeneity of the western Lithuanian *Tilia cordata* populations as (a) possible effects of provenance transfer, especially in the former Eastern Prussia, where different management regime and seed collection sources are expected (region 1B; Kembrytė et al., 2021), (b) presence of large manor parks in Palanga, Kretinga, and Plunge, as source of nonlocal unique genotypes, and (c) the highest number of separate river pools (water networks), assuming *Tilia cordata* within a stream network conserves distinct gene pool in longer temporal perspective. The number of separate river pools in western highland (region 1A), coastal lowland (1B) mid‐northern (2A), mid‐southern (2B), northeastern (3), and southeastern (3) Lithuania is 7, 3, 2, 4, and 3, respectively. In general, presence of environmental heterogeneity and microhabitat diversity helps forming gene pool diversity and so strengthening species for challenges of climate change (Hampe & Petit, 2005; Lobo et al., 2018).

In our study, the Bayesian clustering was more helpful in revealing these geographically consistent genetic structures than the barrier detection by Monmonier's algorithms. As discussed by Safner et al. (2011), the algorithms designed to detect gradients of significant change in allele frequencies are less efficient with highly heterogeneous material as they tend to delineate the outstanding populations rather than the large‐scale geographical structures. In our material, however, the BARRIER runs were useful not in finding where the barriers are but where they are not present given the settings to find any possible barriers (large number of bootstraps and allowing many barriers, Figure S3). In agreement to the above, the weakest barriers in our material were found among rather homogeneous populations in the midland lowland (region 2A and 2B, Figure S3).

The *Tilia cordata* populations on the poor and dry soils of the eastern highland (region 3) were most differentiated in Lithuania. There could be several reasons for that (a) adaptation to cooler climate, drier and poorer sites in this region, (b) gene flow from the east as discussed above on high rare allele frequency in this region, (c) stronger genetic drift effects due to relatively stronger fragmentation caused by to higher urban pressure and harsher environment for *Tilia* in this region.

Our findings indicate that escapes from urban sources may lead to a reduction of genetic diversity in *Tilia cordata* populations. Such diversity reduction is especially undesirable at the northern edge of species advancement, where fragmentation of *Tilia cordata* gene pool is still profound and the environment for sexual reproduction is less favorable (Logan et al., 2019; Pigott et al., 1980). Measures to reduce spreading of *Tilia* sp. from urban sources must be reviewed (like disposal of leaves in the forests). Conservation efforts could consider capturing the genetic diversity of *Tilia cordata* within each of the western, midland, and eastern genetic clusters in Lithuania by identifying autochthonous stands. Asexual reproduction, presence of old‐growth trees, and variable age structures could be considered as the main criteria for the autochthonous populations. The commercial value of *Tilia cordata* trees can be one but not a decisive factor for selecting the conservation stands. Natural population management should favor preservation of old‐growth trees of *Tilia cordata* in mixed forest stands (Figure S6). These trees are the best shelter for bee nests and so successful pollination so needed to produce vital seeds and successful spreading of *Tilia cordata*.

## 5 CONCLUSIONS

Natural populations of *Tilia cordata* in Lithuania retained high levels of genetic diversity, indicating no marked genetic drift effects nor large‐scale problems with inbreeding in the naturally spreading gene pool of *Tilia cordata* in Lithuania as a whole. However, the geographical distribution of the genetic diversity varies within the country.

Weak genetic differentiation among the *Tilia cordata* populations in Lithuania implies common ancestry, absence of strong adaptive gradients, and effective genetic exchange among the *Tilia cordata* populations in the country. A hypothesis on riparian networks as gene flow mediators in *Tilia cordata* was raised based on results of this study.

The geographical patterns of genetic variation observed in our study are likely to reflect the compound effects of habitat fragmentation and suitability for *Tilia cordata*, gene flow patterns, and genetic imprints of recent and historical urban escapes. Due to the variable importance of these factors in different regions of Lithuania, *Tilia cordata* is structured into specific genetic clusters for western, midland, and eastern ecoclimatic regions of Lithuania.

## CONFLICT OF INTEREST

The authors declare no conflict of interest.

## AUTHOR CONTRIBUTION

**Darius Danusevičius:** Conceptualization (equal); Formal analysis (equal); Funding acquisition (equal); Methodology (equal); Software (equal); Supervision (equal); Writing‐original draft (equal). **Ruta Kembryte:** Investigation (equal). **Jurata Buchovska:** Investigation (equal). **Virgilijus Baliuckas:** Conceptualization (equal); Funding acquisition (equal); Methodology (equal); Resources (equal). **Darius Kavaliauskas:** Investigation (equal).

## Supporting information

Supplementary Material

## Data Availability

The data on the location of the populations and microsatellite genotypes from this study were deposited in Dryad data base with https://doi.org/10.5061/dryad.c866t1g64
