# Supplementary material for: Genetic signature of the natural gene pool of Tilia cordata Mill. in Lithuania: Compound evolutionary and anthropogenic effects
Source: Ecol Evol. 2021 May 1;11(11):6260–75. doi: 10.1002/ece3.7473 (PMC8207418; doi:10.1002/ece3.7473)
Supplement: Supplementary file 1 — Supplementary Material [file ECE3-11-6260-s001.docx]

# SUPPLEMENTARY MATERIAL

Table S1. The microsatellite primer sequences and characteristics. All primers from Phuekvilai and Wolff (2013).

| Primer | Sequence (5' - 3') | Allele size range (bp)* | GeneBank  accession no. |
| --- | --- | --- | --- |
| Tc6 F  Tc6 R | CCATATCTTCTGCCAGTTTTCC  GGACTAATTTCTTCCTTTTATTAGGC | 123-146 | JQ289159 |
| Tc937 F  Tc937 R | AGCCAACCAACTTTTACAATACAG  AGATAAAAGCACATAAATCGATGG | 152-176 | JQ289169 |
| Tc920 F  Tc920 R | AAATGTCTTCAGAGTGACTAGATGG  TGCCTCATTATTCTCCTAATTCTC | 216-247 | JQ289167 |
| Tc943 F  Tc943 R | ATTTCATCTTTCTCTAAAGCCTTG  GGGAAAGCCTGTGTTAGTTTC | 141-143 | JQ289170 |
| Tc8 F  Tc8 R | CGAAGAAACTGTCAAAACAACG  AGCTGGGTTTTAGAGGATAGGG | 147-170 | JQ289161 |
| Tc31 F  Tc31 R | TTTGCAAAGACTACTCCAAGAATC  AAATCGATGGTCAAGAACTAAATC | 196-216 | JQ289164 |
| Tc4 F  Tc4 R | ATTTTAGAATGCCAACCTGCTAAG  TATTGAAGTCCATTTCCAATTGTC | 203-251 | JQ289157 |
| Tc927 F  Tc927 R | AGTCCTCCTGTCAAATGCTG  ATCACACTCGTTTATGACATCTTG | 144-184 | JQ289168 |
| Tc11 F  Tc11 R | AGCTATGAAAGAACTATCAAGAGAAAG  CCCCAAGACATTGCAGTAGAAC | 131-165 | JQ289162 |
| Tc915 F  Tc915 R | ACATCGATTGTATTTCCCTTTAAC  GTTGTATTTTGCCCTTAACATTG | 143-189 | JQ289165 |
| Tc963 F  Tc963 R | CTAACCCCATTCTCTTTAATTCTG  GCTTTCATTTCAGTTTTCCTCTAC | 228-279 | JQ289172 |
| Tc951 F  Tc951 R | TGTTATGACCTCACTTATAACCAAGT  GGGTGAGCTGACAATATAGAAGAG | 151-178 | JQ289171 |
| Tc5 F  Tc5 R | TTTTCATACATTTAGAGACTTTTAGCA  TGCATGATTTGTATGTTTAGGG | 144-181 | JQ289158 |
| Tc7 F  Tc7 R | TTTACTTTTGCCAGTTGTGAGG  CACCTAGAATGCCTCCTATTCG | 231-247 | JQ289160 |

*- allele range from Phuekvilai and Wolff (2013).

Table S2. Results from AMOVA with 3 region structure (soft. Arlequin). The P-value tests were based on 10100 permutations.

| Source | d.f. | Variance % | Fixation indexes | P-value |
| --- | --- | --- | --- | --- |
| Among regions | 2 | 0.21 | 0.002 | 0.04307+-0.00218 |
| Populations within regions | 20 | 3.54 | 0.035 | 0.00000+-0.00000 |
| Trees within populations | 1063 | 96.26 | - | - |
|  |  |  |  |  |
| Among populations ^1^ | - | - | 0.037 | 0.00000+-0.00000 |

^1^- no region structure.

| 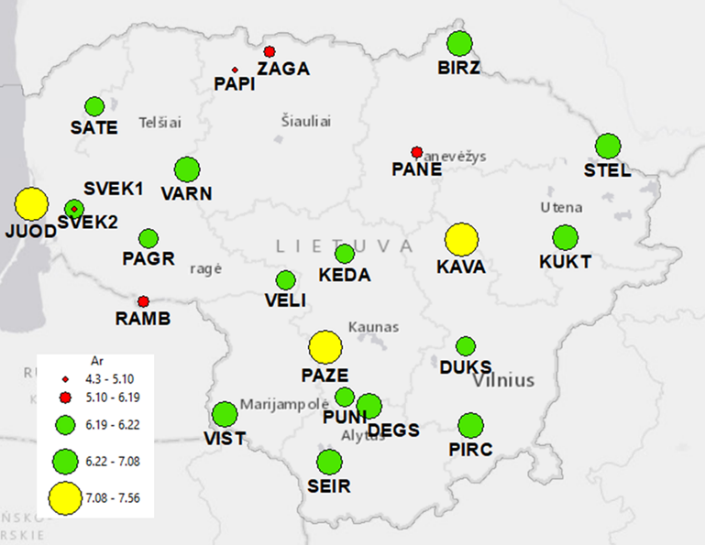 | 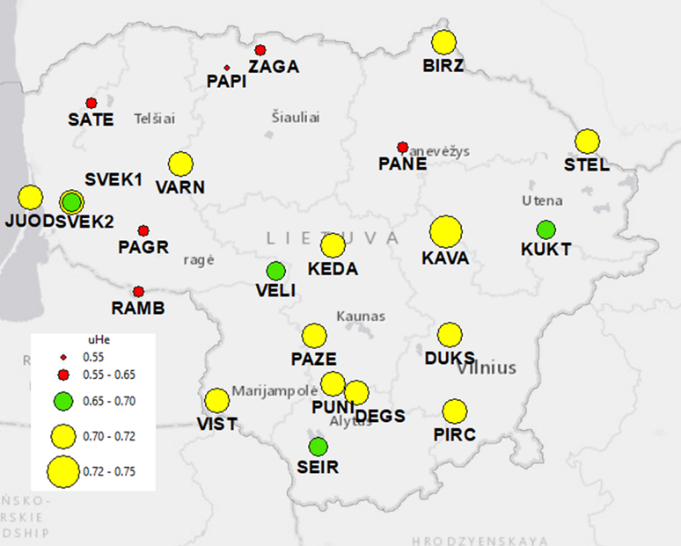 |
| --- | --- |
| 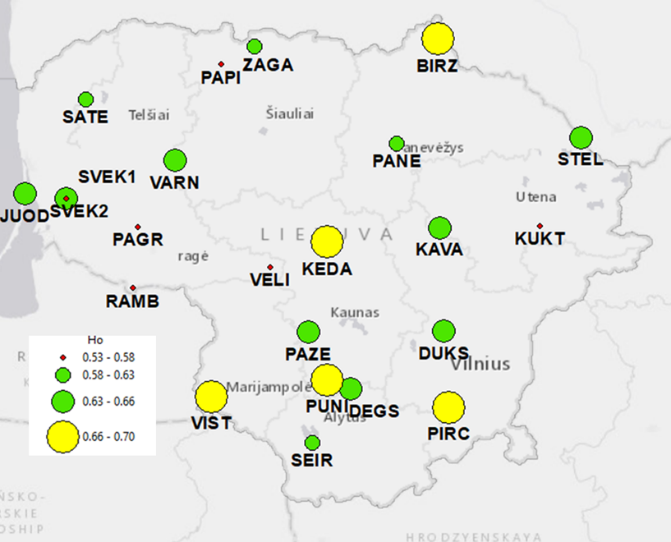 | 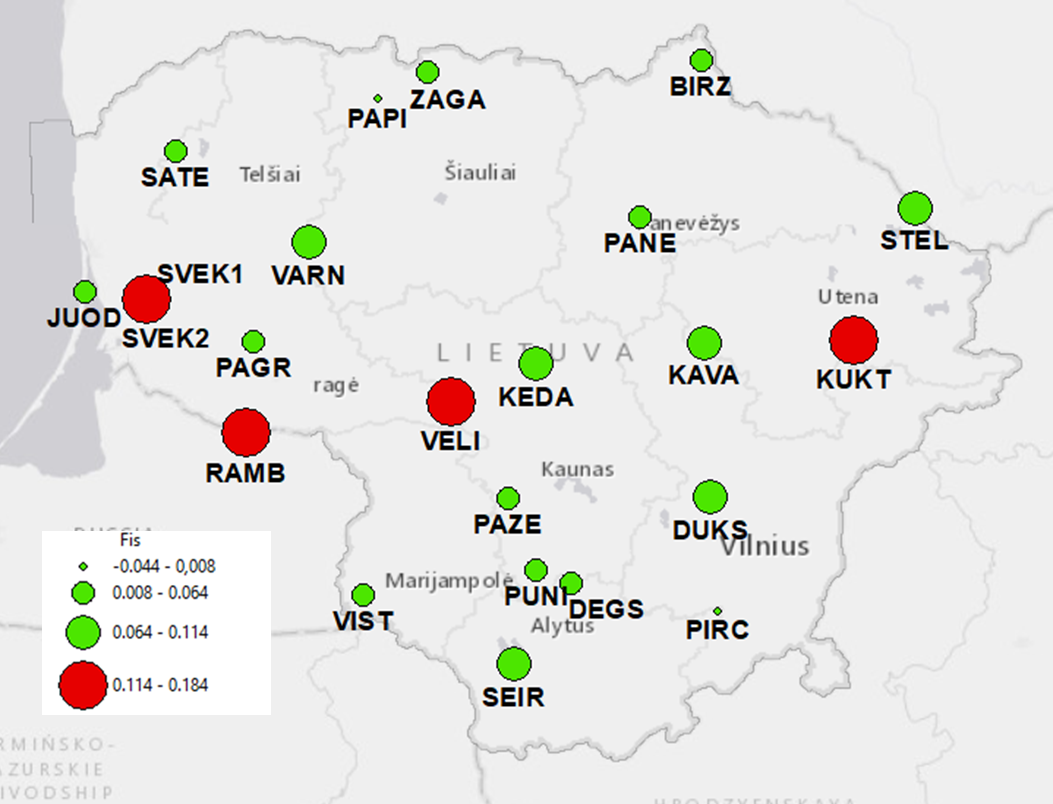 |
| 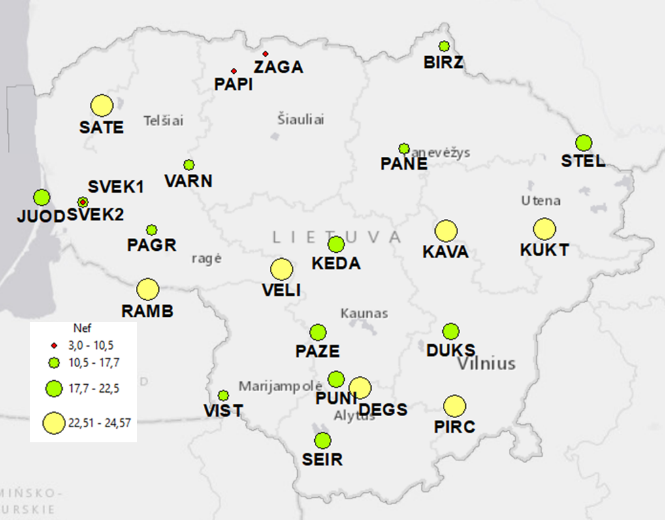  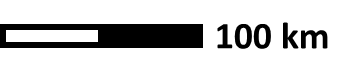 | 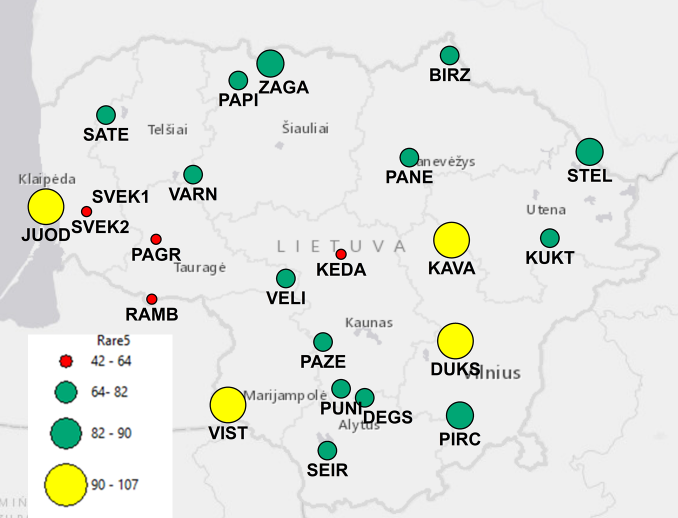 |
| Fig. S1. Geographical distribution of within-population genetic diversity parameters. The topmost row of maps: allelic richness (A_r_, ratification basis 17) and expected heterozygosity (H_e_), the middle row of maps: observed heterozygosity (H_o_) and inbreeding coefficient (F_is_) and the bottom row: effective population size N_ef_ and number of rare alleles below 5% frequency per population (Rare5). | |


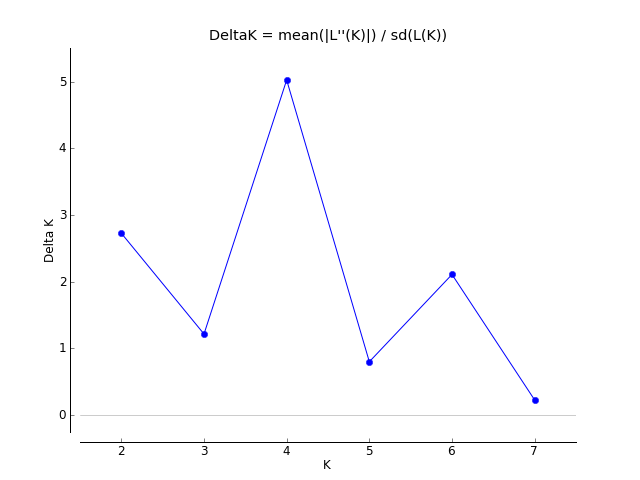


Fig. S2. Output from STRUCTURE HARVESTER software on the optimal deltaK value.

| 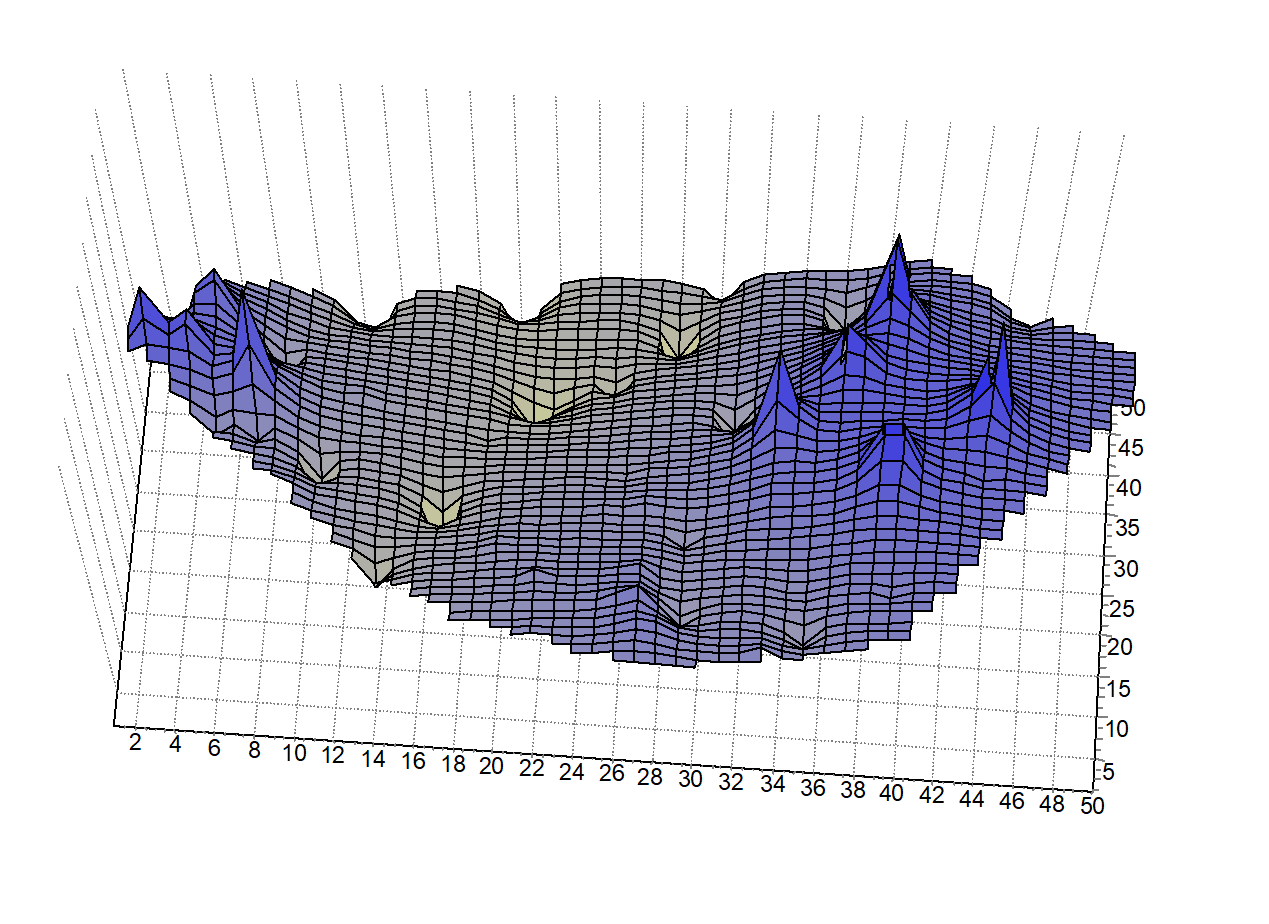 | 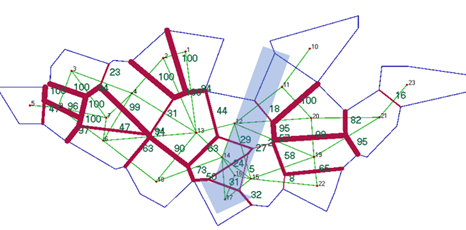 |
| --- | --- |
| Fig. S3. Surface plot of the interpolated Nei’s et al. (1983) genetic distances among *Tilia cordata* populations in Lithuania (AiS soft., left). Barriers of significant allele frequency shifts estimated by the Monmonier’s algorithm on Nei et al. (1983) genetic distances and plotted on the Delauney triangulation graph (BARRIER soft right). The thick red lines are the 10 first genetic barriers with the number of bootstrapped distance matrixes out of 100 returning a particular barrier. The broad arrow indicates absence of significant barriers. | |


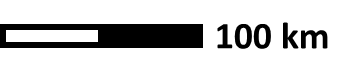

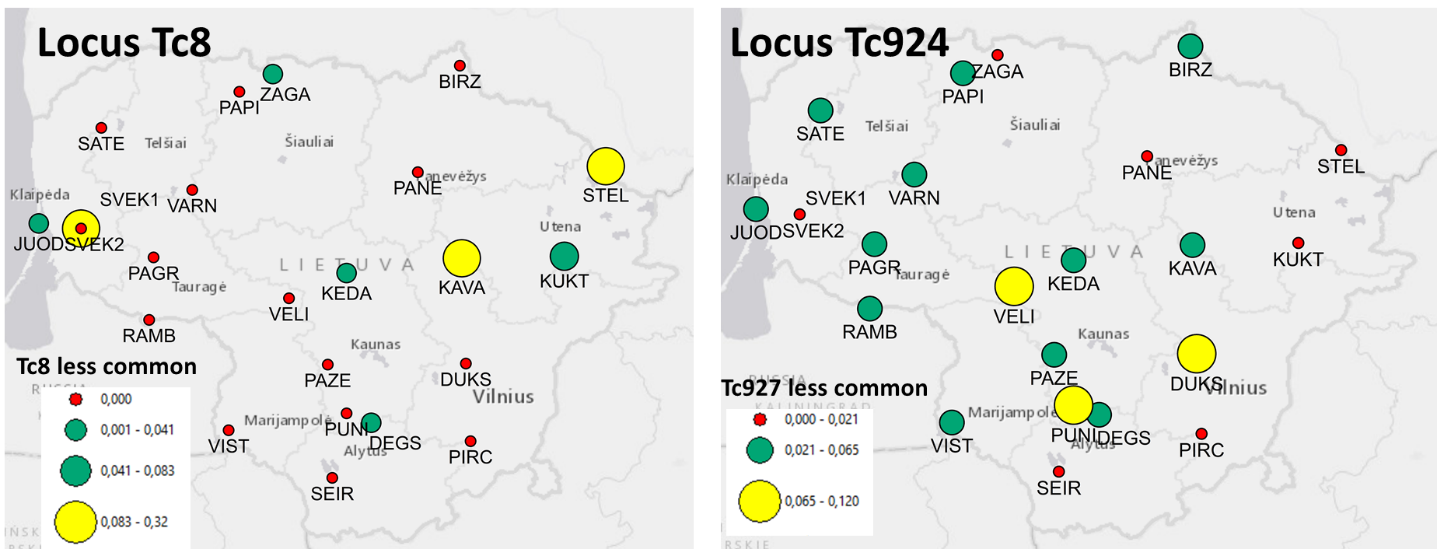


Fig. S4. Possible hybridization signature between *T. cordata* and *T. platyphyllos* as revealed by the geographical distribution map of the less common frequency alleles for the locus Tc927 (4 least common alleles displayed out of 9, where the most common allele of 141 bp occurred at frequency of 0.86). The locus Tc927 was reported to discriminate well between the *T. cordata* and *T. platyphyllos* (Logan et al. 2015).


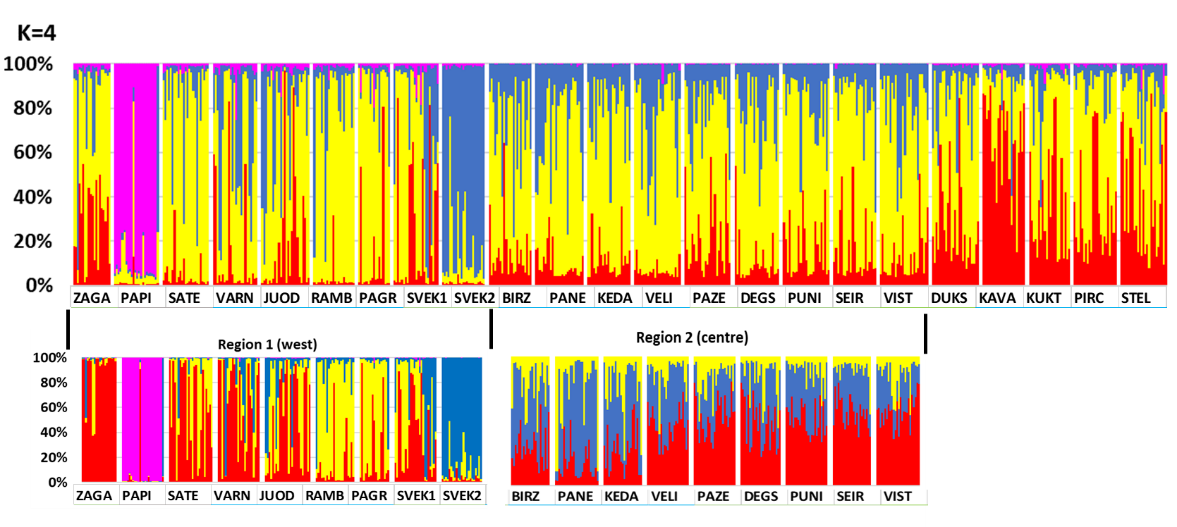


Fig. S5. Attempt to refine the genetic structure within region 1 (west) and within region 2 (midland). Results of STRUCTURE Bayesian clustering analysis illustrating the membership proportions of (a) all *Tilia cordata* individuals into K=4 clusters (upper plot, taken from Fig. 3 for reference), (b) separate clustering on subset of individuals from western region 1 (the bottom left histogram) and (c) subset of individuals from the midland lowland region 2 (bottom, center). Population ids. age given on the X axis. The topmost histogram is the K=4 structure obtained by the analyzing all the material (presented in Fig. 3).


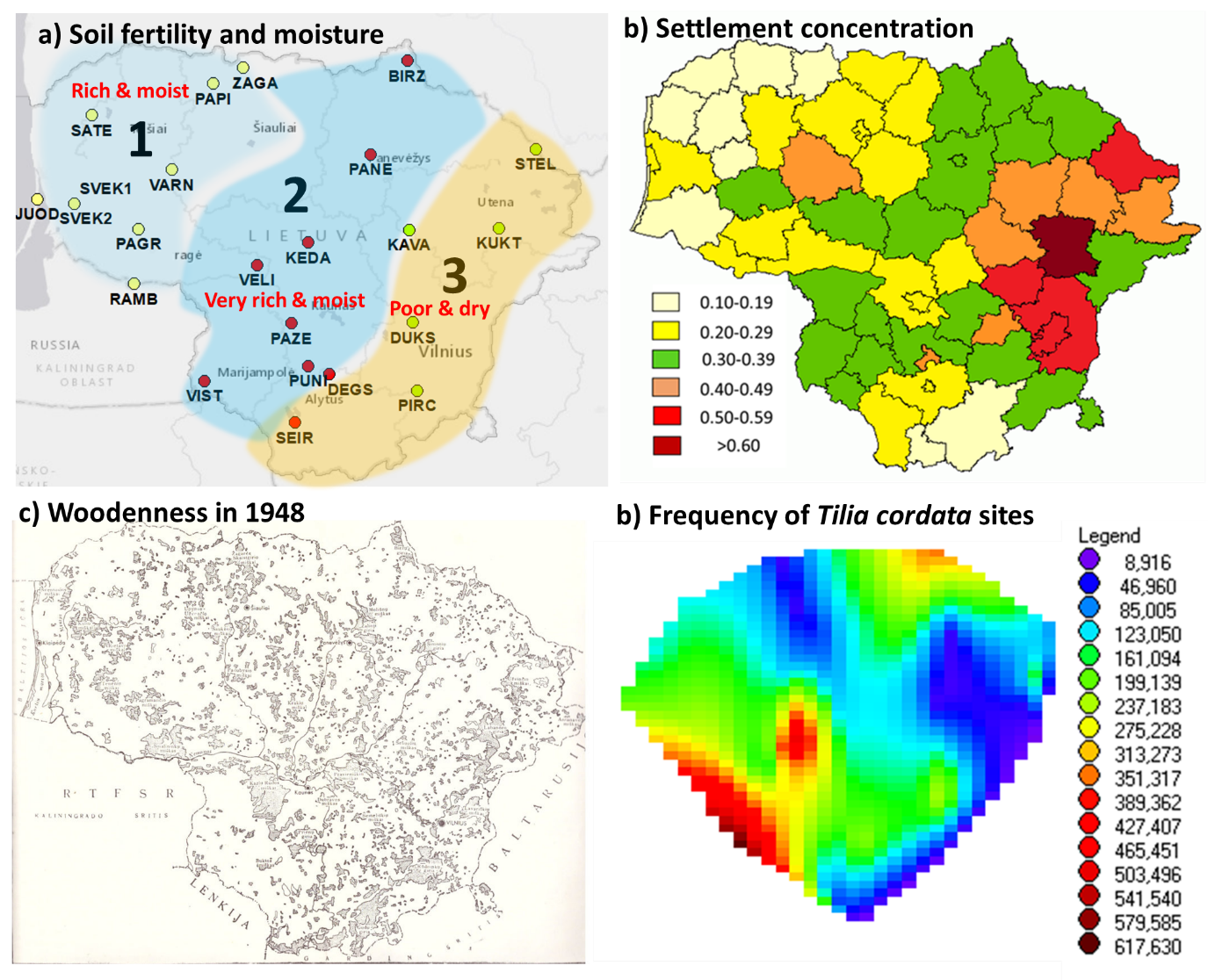


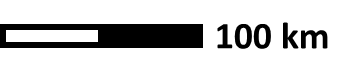


Fig. S6. Landscape differentiation maps of Lithuania helping to interpret the *Tilia cordata* genetic structure obtained in our study. (a) a simplified soil moisture and fertility map, (b) concentration of urban territories by municipality: concentration index = is number of settlements divided by the area (km^2^) of a municipality; (c) Forest tract network during the lowest woodenness of 19.8 % in 1948 in Lithuania (Basalykas, 1965); (d) distribution *Tilia cordata* dominated sites (are in ha of sites with >50% of *Tilia cordata*) within each forest management unit of ca. 40 t. ha in size (forest inventory data from 2010).

A. Basalykas, A. (1965). Lietuvos TSR fizinė geografija II (Physical geography of Lithuanian SSR). Mintis, 496 p. (n Lithuanian)


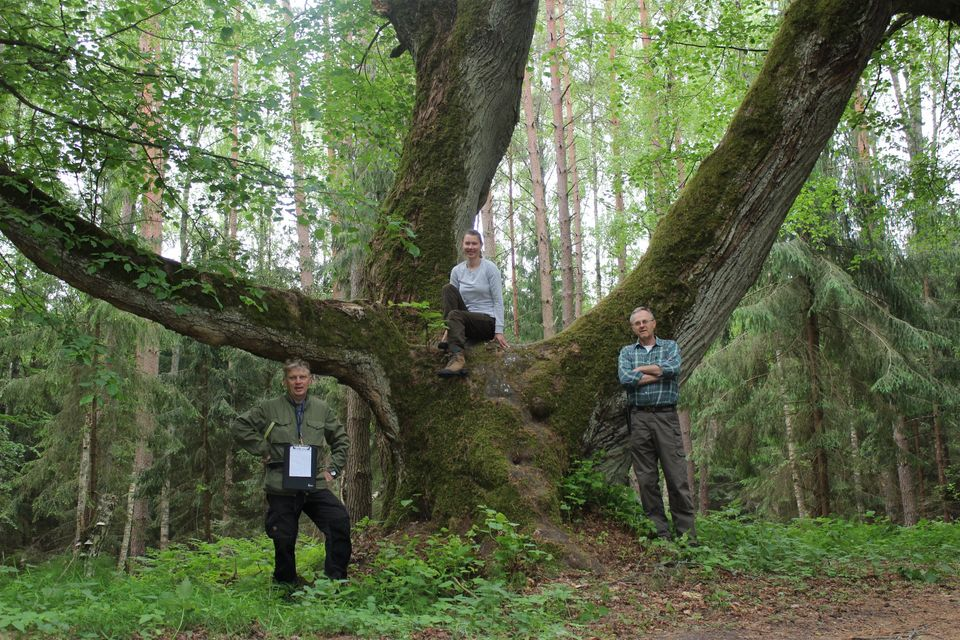


Fig. S7. The old growth *Tilia cordata* trees are the best shelter for bee nests and their presence is very important for genetically healthy spreading *Tilia cordata* populations. Authors of this paper (left to right Darius. D, Ruta K. and Virgilijus B.) in one of the autochthonous populations of *Tilia cordata* of PUNI located in center of the midland lowland of Lithuania, where the *Tilia cordata* dominates the site had this 300 cm diameter *Tilia cordata* tree at its center.
